# Intrahippocampal Infusion of Crotamine Isolated from *Crotalus durissus terrificus* Alters Plasma and Brain Biochemical Parameters †

**DOI:** 10.3390/ijerph111111438

**Published:** 2014-11-05

**Authors:** Rithiele Gonçalves, Liane S. Vargas, Marcus V. S. Lara, Angélica Güllich, Vanusa Mandredini, Luis Ponce-Soto, Sergio Marangoni, Cháriston A. Dal Belo, Pâmela B. Mello-Carpes

**Affiliations:** 1 Physiology Research Group, Graduate Program on Biochemistry, Federal University of Pampa (UNIPAMPA), P.O. Box 118, 97500-970, Uruguaiana, RS, Brazil; E-Mails: rithiele.gpfis@gmail.com (R.G.); lianeevargas@gmail.com (L.S.V.); marcus1192@hotmail.com (M.V.S.L.); angelicagullich@hotmail.com (A.G.); vanusa_manfredini@yahoo.com.br (V.M.); 2 Laboratório de Química de Proteínas (LAQUIP), Department of Biochemistry, Institute of Biology, State University of Campinas (UNICAMP), P.O. Box 6109, 13083-970 Campinas, SP, Brazil; E-Mails: poncesot@unicamp.br (L.P.-S.); marango@unicamp.br (S.M.); 3 Neurobiology and Toxinology Group (LANETOX), Federal University of Pampa (UNIPAMPA), 97300-000, São Gabriel, RS, Brazil; E-Mail: charistonbelo@unipampa.edu.br

**Keywords:** snake venom, toxicity, inflammation, oxidative parameters, brain, plasma

## Abstract

Crotamine is one of the main constituents of the venom of the South American rattlesnake *Crotalus durissus terrificus*. Here we sought to investigate the inflammatory and toxicological effects induced by the intrahippocampal administration of crotamine isolated from *Crotalus* whole venom. Adult rats received an intrahippocampal infusion of crotamine or vehicle and were euthanized 24 h or 21 days after infusion. Plasma and brain tissue were collected for biochemical analysis. Complete blood count, creatinine, urea, glutamic oxaloacetic transaminase (GOT), glutamic pyruvic transaminase (GPT), creatine-kinase (CK), creatine kinase-muscle B (CK-MB) and oxidative parameters (assessed by DNA damage and micronucleus frequency in leukocytes, lipid peroxidation and protein carbonyls in plasma and brain) were quantified. Unpaired and paired *t*-tests were used for comparisons between saline and crotamine groups, and within groups (24 h *vs.* 21 days), respectively. After 24 h crotamine infusion promoted an increase of urea, GOT, GPT, CK, and platelets values (*p* ≤ 0.01), while red blood cells, hematocrit and leukocytes values decreased (*p* ≤ 0.01). Additionally, 21 days after infusion crotamine group showed increased creatinine, leukocytes, TBARS (plasma and brain), carbonyl (plasma and brain) and micronucleus compared to the saline-group (*p* ≤ 0.01). Our findings show that crotamine infusion alter hematological parameters and cardiac markers, as well as oxidative parameters, not only in the brain, but also in the blood, indicating a systemic pro-inflammatory and toxicological activity. A further scientific attempt in terms of preserving the beneficial activity over toxicity is required.

## 1. Introduction

Snakebites are a public health issue in tropical countries [1]. In Brazil, the South American rattlesnake *Crotalus durissus Terrificus* (*Cdt*) accounts for 10% of snakebites and constitutes a medical problem in the country [2]. Studies with the rattlesnake venoms are carry out mainly to understand the mechanism of action of proteins present in these poisons in order to improve the treatment against the snakebite accidents. However, toxic components of the *Cdt* venom, as crotamine, has shown properties with potential for therapeutic purposes [3,4,5]. 

Crotamine, a non-enzymatic polypeptide myotoxin, basic character, composed by 42 amino acid residues [6], is one of the components of the *Cdt* venom. This toxin induces depolarization of the skeletal muscle membrane by increasing the permeability to sodium ions (Na^+^), suggesting its binding to voltage-gated Na^+^ channels at the sarcolemma [7]. Despite the potential interaction of crotamine with sodium channels, some opposing studies suggested another target for the toxin biological activity [8]. In the central nervous system (CNS) of rats, crotamine improves the basal release of acetylcholine and dopamine [9], which are neurotransmitters strikingly related to the mnemonic process [10,11]. 

Considering the crotamine pharmacology, recent studies have shown possible therapeutic applicabilities for this toxin. Hernandez-Oliveira e Silva *et al.* [4] showed the benefits of using crotamine in the treatment of myasthenia gravis, an autoimmune disease characterized by a dysfunction at the neuromuscular junction, resulting in episodes of muscle weakness and abnormal fatigue [12]. In their study, animals treated with crotamine showed progressive and sustained muscle contractions, as well as an improvement in exercise tolerance [4]. The application of crotamine induced a decrease in the episodes of muscle fatigue compared to animals treated with neostigmine, a common clinical agent used in the myasthenia treatment [4]. Recently, crotamine was proven to be a cell-penetrating peptide that can mediate the drug delivery, being cytotoxic to neoplasic cells [3].

We recently showed that intrahippocampal infusion of a single dose of crotamine (1 µg/µL; 1 µL/side) improves after-learning persistence of object recognition and aversive memory [5]. This result reinforces the potential therapeutic application of crotamine as a novel therapeutic agent to treat diseases related to the CNS function, especially in situations in which the loss of cognitive function is involved, such as dementias. Apart from the biotechnological perspectives involving crotamine, the neurotoxicity induced by the *Cdt* venom components at central and peripheral nervous system has been characterized [13,14,15]. Thus, despite the wealth of possibilities in terms of the possible therapeutic interactions of crotamine at the nervous system, there is few data related to its safety assessment evaluation. The aim of this work was to investigate the influence of crotamine on inflammatory and toxicological parameters after intrahippocampal administration. 

## 2. Methods 

### 2.1. Animals

Adult male Wistar rats (3 months old) were bought at a registered vivarium. They were housed four per cage and maintained under controlled light and environmental conditions (12 h light/12 h dark cycle at a temperature of 23 ± 2 °C and humidity of 50 ± 10%) with food and water *ad libitum*. All experiments were conducted in accordance with the “Principles of laboratory animal care” (NIH publication No. 80-23, revised 1996) and with the guidelines established by the Institutional Animal Care and Use Committee of the Local Institution (IRB #0442012), ensuring that animal number and suffering were kept to a minimum.

**Figure 1:**
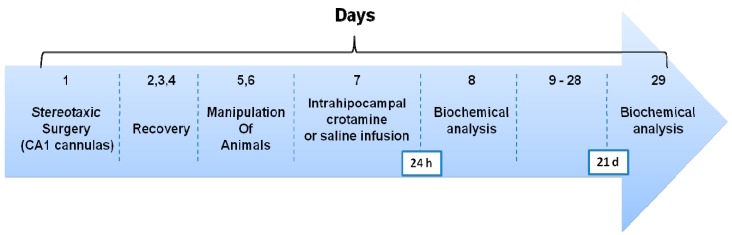
Schematic representation of the experimental procedures. Rats were submitted to a stereotaxic surgery to implant cannulas in the CA1 region of the hippocampus. After 3 days of recovery, they are manipulated for 2 days to adapt to the experimenter. Following the procedures of cannulas’s implantation and recovery the rats in the saline-group received an intrahippocampal infusion of 1 µL/side of vehicle (saline), and rats in the crotamine-group 1 µL/side of crotamine (1 µg/µL). Afterward, six animals from each group were euthanized twenty four hours and six twenty one days after crotamine or saline intrahippocampal infusion for tissue preparation and biochemical analyses.

To study the systemic effects of single infusion of crotamine in CNS, 24 rats were implanted with chronic bilateral guide cannulas in the CA1 region of the hippocampus and divided into two groups: controls (*n* = 12), which received 1 µL/side of vehicle (saline-group), and crotamine (*n* = 12), which received 1 µL/side of crotamine infusion (1 µg/µL; 1 µL/side; crotamine-group). Afterward, 6 animals from each group were euthanized twenty four hours and 6 twenty one days after crotamine or saline intrahippocampal infusion for tissue preparation and biochemical analyses (Figure 1). 

### 2.2. Drugs

Ketamine and xylazine were purchased from Sigma Aldrich Brazil (São Paulo, SP, Brazil). Crotamine isolated from *Cdt* venom was a reminiscent stock and was supplied by Dr. Sérgio Marangoni from Laboratory of Protein Chemistry (LAQUIP) (UNICAMP, Campinas-SP, Brazil) and was prepared daily by dilution in saline immediately before use. The crotamine dose was chosen based on previous *in vitro* [9] and *in vivo* [5] experiments. Reagents for oxidative parameters analysis were purchased from Sigma (St. Louis, MO, USA), and other reagents used in this study were of analytical grade and obtained from standard commercial supplier.

### 2.3. Surgery and Drug Infusion Procedures

To implant the rats with indwelling cannulas, they were anesthetized with ketamine and xylazine (i.p., 75 mg/kg and 10 mg/kg, respectively) and 27-gage cannulas were placed, stereotaxically aimed at CA1 region of the dorsal hippocampus coordinates (AP-4.2, LL-3.0, VM-2.0 mm), according to Paxinos and Watson atlas [16]. The cannulas were affixed to the head bones with dental cement. Animals were allowed to recover from surgery for 3 days before submitting them to any other procedure. At the time of drug delivery, 30-gage infusion cannulas were tightly fitted into the guides. Infusions (1 µL/side in the CA1 region of the hippocampus) were carried out over 60 s with an infusion pump, and the cannulas were left in place for 60 additional seconds to minimize backflow.

### 2.4. Biochemical Analysis 

Twenty four hours and twenty one days after crotamine intrahippocampal infusion, rats from each group were submitted to euthanasia. It permitted collection of tissue (blood and brain) for biochemical analyses: hematological parameters, cardiac and oxidative markers. Six rats of each group were euthanized in each time.

The complete blood count (CBC) was performed in an automatic counter Cell-Dyn 3200 Hematology Analyzer (Abbott Diagnostic, St Clara, CA, USA). Blood creatinine, urea, glutamic oxaloacetic transaminase (GOT), glutamic pyruvic transaminase (GPT), and creatine-kinase (CK) levels were measured using an automatic analyzer A-25 Biosystems (Biosystems SA, Barcelona, Spain) for *in vitro* diagnostics. Blood creatine kinase-muscle B (CK-MB) was measured using an Architect Abbott for *in vitro* diagnostics. 

The oxidative parameters (DNA damage and micronucleus frequency in leukocytes [17]; lipid peroxidation [18] and protein carbonyls [19]) in plasma and brain were measured using spectrophotometric methods. For brain analyses, the brain was homogenized in 10 volumes of Tris HCl (50 mM, pH 7.4). Afterwards, samples were centrifuged at 2400× g for 20 min, and supernatant were used for assay. 

Lipoperoxidation was evaluated by the thiobarbituric acid reactive substance (TBARS) test [18]. Samples were rapidly homogenized in 50 mM Tris-HCl, pH 7.5 (1/10, w/v), and centrifuged at 2500× g for 15 min. One aliquot of supernatant was incubated at 95 °C for 2 h, and the colour reaction was measured at 532 nm. Results were expressed as nmol of malondialdehyde per mg protein. All plasma and brain biochemical assays were carried out in triplicate.

### 2.5. Statistical Analysis

To compare biochemical markers between saline and crotamine-group unpaired Student *t*-test was used. For comparison of biochemical markers of the same group in different times (24 h and 21 days) a paired Student *t*-test was used. All data were expressed as the mean ± SEM. Differences were considered statistically significant when *p* < 0.01.

## 3. Results 

After euthanasia, blood and brain tissue were collected and prepared for analyses of hematological, cardiac markers and oxidative parameters. Results of hematological and cardiac parameters after 24 h and 21 days of crotamine/saline infusion are present in the Table 1. In the saline-group there were no statistical differences between 24 h and 21 days for all parameters analyzed (data presented as mean ± SEM of the measurements; Table 1 saline-group).

**Table 1:** Results of blood hematological and cardiac parameters. The intrahippocampal infusion of crotamine altered some measurements 24 h and 21 days after infusion. Data are expressed as mean ± SEM.

Blood Hematological and Cardiac Parameters	Saline-Group	Crotamine-Group	
		24 h	21 Days
Creatinine (mg/dL)	0.58 ± 6 × 10^−3^	0.57 ± 5 × 10^−3^	0.83 ± 0.01 ^#,*^
Urea (mg/dL)	45.50 ± 0.89	52.75 ± 1.26 ^*^	34.29 ± 0.30 ^#,*^
GOT (U/L)	120.00 ± 0.56	142.00 ± 2.22 ^*^	266.10 ± 3.32 ^#,*^
GPT (U/L)	73.25 ± 0.98	82.75 ± 2.75 ^*^	94.43 ± 1.11 ^#,*^
CK (U/L)	806.30 ± 4.69	947.00 ± 15.71 ^*^	15 × 10^2^ ± 12.62 ^#,*^
CK-MB (U/L)	22 × 10^2^ ± 19.11	23 × 10^2^ ± 49.86	24 × 10^2^ ± 0.06
Red Blood Cells (106/µL)	7.21 ± 0.01	6.66 ± 0.04 ^*^	7.84 ± 0.31
Hemoglobin (g/dL)	13.58 ± 0.11	13.10 ± 0.16	13.40 ± 0.25
Hematocrit (%)	40.88 ± 0.18	38.60 ± 0.57 ^*^	37.76 ± 0.50 ^*^
Leukocytes (103/µL)	58 × 10^2^ ± 57.90	45 × 10^2^ ± 56.41^*^	93 × 10^2^ ± 253.10 ^#,*^
Platelets (103/µL)	44 × 10^4^ ± 20 × 10^2^	53 × 10^4^ ± 32 × 10^2^ ^*^	89 × 10^4^ ± 16 × 10^2^ ^#,*^

^*^ Statistically significant differences (*p* < 0.01) from saline-group; **^#^** Statistically significant differences (*p* < 0.01) from 24 h crotamine-group.

The analysis of data related to 24 h post-crotamine infusion showed that toxin induced an increase in the values of urea, GOT, GPT, CK and platelets when compared to results from saline-group (*p* < 0.01, Table 1, crotamine-group 24 h). Additionally, toxin infusion induced a decrease in red blood cells, hematocrit and leukocytes values. Twenty-one days post-crotamine infusion we observed that the values of GOT, GPT, CK, and platelets still were increased and remained different than the saline group (*p* < 0.01, Table 1 crotamine-group 21 days). Also, the hematocrit values still were decreased 21 days after crotamine infusion (*p* < 0.01, Table 1 crotamine-group 21 days). For this later group, creatinine and leukocytes values also increased and urea values decreased (*p* < 0.01, Table 1 crotamine-group 21 days). 

Oxidative parameters showed no changes 24 h-post crotamine infusion. However, 21 days-post infusion the values of TBARS (plasma and brain), carbonyl (plasma and brain) and micronucleus were increased in the crotamine-group compared to the saline (*p* < 0.01, Table 2 crotamine-group 21 days). In the case of micronucleus, crotamine-infusion induced a further significant increase when compared to the toxin 24 h group.

**Table 2:** Results of plasma and brain oxidative parameters. The intrahippocampal infusion of crotamine altered oxidative parameters 21 days later. Data are expressed as mean ± SEM.

Plasma and Brain Oxidative Parameters	Saline-Group	Crotamine-Group	
		24 h	21 Days
**TBARS Plasma (nmol MDA/L)**	27.43 ± 0.27	27.43 ± 0.92	50.51 ± 1.37 ^#,*^
**TBARS Brain (nmol MDA/L)**	96.13 ± 1.32	97.73 ± 3.62	143.30 ± 4.34 ^#,*^
**Carbonyl Plasma (nmol carbonyl/mg protein)**	0.01 ± 2 × 10^−4^	0.01 ± 4 × 10^−4^	0.02 ± 6 × 10^−4 #,*^
**Carbonyl Brain (nmol carbonyl/mg protein)**	0.01 ± 3 × 10^−4^	0.01 ± 5 × 10^−4^	0.02 ± 7 × 10^−4 #,*^
**Micronucleus (% frequency)**	0.75 ± 0.13	1.00 ± 0.21	1.87 ± 0.19 ^#,*^

^*^ Statistically significant differences (*p* < 0.01) from saline-group. **^#^** Statistically significant differences (*p* < 0.01) from 24 h-post crotamine-group.

## 4. Discussion

There are about 3000 species of snakes in the world, with 20% of them considered poisonous [20]. Annually 19,000 to 22,000 snake accidents occur in Brazil [21]. Most of these accidents are due to the *Bothrops* and *Crotalus* genus [22], so it is crucial understand the systemic mechanisms involved in the toxicity of snake venoms to improve the treatment of the snakebite victims. In this work we show the toxic aspects involved with the intrahipocampal infusion of crotamine, especially those related to the hematological and biochemical parameters. The aspects related to the safety assessment of crotamine are discussed herein.

Crotamine is a strong basic polypeptide myotoxin consisting of 42 amino acid residues present in the venom of the *Crotalus durissus terrificus*, the South American rattlesnake. Studies have investigated its potential therapeutic applicability, although there is a lack of data related to the safety assessment of this toxin [4,5]. Mello and Cavalheiro [15] studied the induction of neuropathological changes by the infusion of *Cdt* venom at hippocampus, but they did not investigate the toxicological aspects related to the intrahippocampal infusion of *Cdt* venom. In this work, we have demonstrated that a single intrahippocampal infusion of crotamine induced acute and chronic alterations in hematological and cardiac parameters in blood and promoted sub-acute oxidative stress in plasma and brain. It is worth mentioning that we recently demonstrated, that a single intrahippocampal infusion of crotamine can promote memory persistence [5], highlighting that crotamine could be a potential pharmacological tool, especially in case of diseases that involve memory persistence deficits, as Alzheimer disease. We now showed that crotamine infusion induced alterations in creatinine, CK, leukocytes, platelets, urea, GOT, GPT, red blood cells and hematocrit in the blood; and TBARS, carbonyl and micronucleus in the plasma and brain tissue. 

Myotoxins of snake venoms are currently classified into three main groups that constitute structurally distinct protein families. Group 1 includes ‘small’ myotoxins (*i.e.*, *Crotalus durissus terrificus* crotamine, *Crotalus v. viridis* myotoxin a); group 2 includes cardiotoxins; and, group 3 includes PLA_2_ myotoxins [23]. The biological activity of myotoxins of snake venoms involves irreversible damage to skeletal muscle fibers (myonecrosis) when injected into higher animals. Some myotoxins act locally, damaging muscle fibers at the site of injection and its surroundings; others act systemically, causing muscle damage at distant sites [24]. Despite the muscular damage (myotoxicity), these toxins can also induce other biological activities, which include neurotoxicity and effects such as anticoagulation, alterations in platelet aggregation, hypotension, hemolysis and edema [24]. Most of the myotoxins involved in deleterious activities against biological tissues are related to the group of PLA_2_ myotoxins. These proteins have evolved from an ancestral PLA_2_ with digestive function and display a range of enzymatic turnover values similar to those digestive enzymes found in pancreatic secretions. 

Despite their weak myotoxic potency, the cellular alterations induced by crotalic venoms are mostly related to the activity of non-neurotoxic PLA_2_ neurotoxins, [23], mainly because their abundance in the whole venom. In the case of crotamine, no enzymatic (PLA_2_) profile is observed, and its pharmacological activity is related to the activation of sodium influx at muscle cells [25] and the blockage of voltage gated potassium channels (Kv) at nerve tissues [26]. At nerve terminals, the activation of voltage-gated sodium channels and/or blockade of Kv by animal neurotoxins ultimately cause increase in the influx of calcium to the cell cytosol [27]. In this regard, it is stated that the influx of Ca^2+^ or Na^+^ ions caused even by subcytolytic concentrations of a snake toxins (or other membrane-active factors), could initiate a variety of intracellular processes, including the activation of endogenous cellular lipases [23]. Indeed, membrane-active peptides of synthetic or natural origin, free of PLA_2_ contamination, which are not catalytic, induce breakdown of phospholipids and production of free fatty acids in skeletal muscle cell cultures, which are compatible with an activation of endogenous phospholipase C enzymes [24]. 

Therefore, we suggest that calcium homeostasis disturbance induced by intrahippocampal infusion of crotamine can account for acute and chronic alterations in hematological and cardiac parameters. This reflects the high affinity of these natural molecules with biological tissues and the evolutionary ability to diffuse into different organ systems of the victims. Sousa-e-Silva *et al.* [28] showed that an endovenous administration (i.p.) of *Cdt* venom in dogs increased CK levels; similar effect was observed in our experimental conditions, following intrahippocampal infusion of crotamine. The increase in the CK levels is generally associated with muscle damage, and can be reinforced by the calcium homeostasis disturbance. Here the intrahippocampal infusion of crotamine also decreased the leukocytes number after 24 h, but increased it after 21 days after the crotamine infusion. A reasonable explanation for this contradictory modulation is that part of the reminiscent crotamine-induced inflammatory process is detectable only in the chronic analysis, but not in the acute protocol. Crotamine-infusion also induced an increase in the platelet number. Alterations in the platelet number following snake venoms administration is a common fact [29]. Hemorrhagic toxins that cause local blood flow impairment, ischemia, and secondary myonecrosis [30], of slow onset, would be considered as indirect myotoxic factors in snake venoms [23]

In addition, 21 days after intrahipocamppal infusion of crotamine we observed the increase of urea levels, which is commonly associated with acute renal lesions [28]. GPT and GOT were also elevated in rats receiving infusion of crotamine. The alterations in the urea levels provided further information about the liver status, revealing a potential hepatotoxic activity of crotamine [31]. The white blood cells, leukocytes, were reduced in the acute evaluation following crotamine infusion, as expected [32]. Furthermore, the significant alterations in the values of creatinine, urea, GOT, GPT, CK, hematocrit, leukocytes and platelets still were present 21 days after crotamine infusion, revealing the irreversible kidney, liver and heart long-term damage and the triggering of a prominent chronic inflammatory response. 

Another important contribution of our work was the verification of the increase in the oxidative stress parameters followed 21 days after crotamine infusion. This activity was evidenced both in the brain and blood tissues and also reflect the long-term alterations induced by crotamine. The oxidative stress occurs when there is an imbalance between the generation of oxidant compounds and antioxidant defense systems, and by the excessive production of reactive oxygen species (ROS) that serve as a regulatory process of the biological homeostasis, but that, in excess, becomes harmful [33]. Da Silva *et al.* [34] demonstrated that different doses of *Cdt* venom increases lipid peroxidation in the liver of mice, corroborating our findings. The increase in lipid peroxidation causes modifications in proteins, DNA and cell membrane [35], because the fatty acids present in these structures are susceptible to peroxidation [36]. In this sense, Dal Belo *et al.* [13] showed the ability of the ethanol extract of *Hypericum brasiliense* to improve the cell viability of hippocampal tissue treated with *Cdt* venom. The latter ascribed to quercetin the protective actions of the vegetal extract, a compound that may be the key to attenuate the oxidative stress caused by crotamine in further pharmacological studies.

Our work is also relevant because it brings to light contradictory aspects related to the toxicology of crotamine. For example, Pereira *et al.* investigated the selectivity of crotamine to tumor cells and showed that in lower doses (10 μM) this toxin is harmless to normal cells [37]. The authors also showed that mice treated for 21 days with crotamine (1 μg/100 μL–2 µM) did not show significant alterations on kidney and liver function, as well as in the blood elements [37]. On the contrary, our study demonstrated that the intrahippocampal infusion of crotamine induced blood parameter alterations and oxidative stress. These differences can be associated with the route of toxin administration, which can be peripheral or via direct CNS injection. Boni-Mitake demonstrated that the intraperitoneal administration of crotamine was able to reach the liver, kidney, skeletal muscle and, to a lesser extent the lungs and spleen of mice [38]. The same authors also verified the native and irradiated crotamine biodistribution after intraperitoneal injection and demonstrated that both forms have hepatic and renal clearance metabolism and have affinity for the skeletal muscle, but do not cross the blood brain barrier [39]. Our experimental design revealed an important aspect about the pharmacokinetics of crotamine, demonstrating the ability of this toxin to cross the blood brain barrier, as argued by other authors that verified that this toxin is able to cross the blood brain barrier and exert CNS effects [6,14]. Notwithstanding, this assumption is valid as, in our case, a single intrahippocampal infusion of crotamine (1 μL/side) was able to alter blood hematological parameters and cardiac markers, as well as brain and plasma oxidative parameters, indicating systemic inflammatory and toxicological effects.

## 5. Conclusions 

Intrahippocampal infusion of crotamine induces toxicity and systemic inflammatory responses in peripheral tissues, proving its ability to cross the blood brain barrier. Therefore, it is necessary to find alternatives in order to decrease crotamine toxicity. Finally, considering the previous studies revealing the therapeutical potential of crotamine upon treatment of cognitive impairments, further studies shall be conducted to improve the knowledge about crotamine biodistribution and systemic toxicity.
